# Early Drought-Responsive Genes Are Variable and Relevant to Drought Tolerance

**DOI:** 10.1534/g3.120.401199

**Published:** 2020-03-11

**Authors:** Cheng He, Yicong Du, Junjie Fu, Erliang Zeng, Sunghun Park, Frank White, Jun Zheng, Sanzhen Liu

**Affiliations:** * Department of Plant Pathology, Kansas State University, Manhattan, KS 66506; ** Department of Horticulture and Natural Resources, Kansas State University, Manhattan, KS 66506; † Hunan Agricultural Technology Extension Station, Changsha, Hunan 410000, China; ‡ Institute of Crop Science, China Academy of Agricultural Science, Beijing 100081, China; § University of Iowa, Division of Biostatistics and Computational Biology, University of Iowa, IA 52242; †† Department of Plant Pathology, University of Florida, Gainesville, FL 32611

**Keywords:** *Zea mays*, drought, transcriptomics, time-series, small RNA

## Abstract

Drought stress is an important crop yield limiting factor worldwide. Plant physiological responses to drought stress are driven by changes in gene expression. While drought-responsive genes (DRGs) have been identified in maize, regulation patterns of gene expression during progressive water deficits remain to be elucidated. In this study, we generated time-series transcriptomic data from the maize inbred line B73 under well-watered and drought conditions. Comparisons between the two conditions identified 8,626 DRGs and the stages (early, middle, and late drought) at which DRGs occurred. Different functional groups of genes were regulated at the three stages. Specifically, early and middle DRGs display higher copy number variation among diverse *Zea mays* lines, and they exhibited stronger associations with drought tolerance as compared to late DRGs. In addition, correlation of expression between small RNAs (sRNAs) and DRGs from the same samples identified 201 negatively sRNA/DRG correlated pairs, including genes showing high levels of association with drought tolerance, such as two glutamine synthetase genes, *gln2* and *gln6*. The characterization of dynamic gene responses to progressive drought stresses indicates important adaptive roles of early and middle DRGs, as well as roles played by sRNAs in gene expression regulation upon drought stress.

Drought is a major environmental stress constraining crop productivity. Maize, as a staple food and forage crop, suffers approximately 15–20% of grain yield losses due to drought. Losses are projected to be higher as water limitation becomes greater owing to urbanization, industrialization, and climate changes (Sayadi Maazou *et al.* 2016; Easterling *et al.* 2000; Christensen *et al.* 2007; Taylor *et al.* 2012). Many crops are not well-adapted to drought, and crop performance under drought conditions may be improved with enhanced understanding of drought responsive mechanisms. Many changes in physiological pathways are involved in responses to drought stress (Bohnert *et al.* 1995; Xiong and Zhu 2002). Physiological changes are driven by transcriptional regulation (Wilkins *et al.* 2010; Baerenfaller *et al.* 2012; Harb *et al.* 2010), and, with the consequential changes in gene expression, result in alternation of protein and metabolite abundance to configure drought defensive mechanisms (Shinozaki and Yamaguchi-Shinozaki 2007; Ozfidan *et al.* 2012; Cruz de Carvalho 2008). Indeed, many drought responsive genes are transcription factors (TFs) that regulate gene expression and signal transduction in stress responses (Shinozaki and Yamaguchi-Shinozaki 1997; Ingram and Bartels 1996). Overexpression of some TFs, including DREB2A, NF-YB, ERF, and NAC, was found to enhance drought resistance in multiple plant species (Sakuma *et al.* 2006; Nelson *et al.* 2007; Chen *et al.* 2008; Quan *et al.* 2010; Tang *et al.* 2012). Non-TF genes, including the gene *ZmVPP1* for a vacuolar-type H(+) pyrophosphatase, are known to be involved in drought resistance (Wang *et al.* 2016).

Phytohormones also play critical roles in drought responses and can regulate gene expression of their responsive genes through directly interacting with gene promoters (Guilfoyle *et al.* 1998; Santner and Estelle 2009; Peleg and Blumwald 2011). One of phytohormones, abscisic acid (ABA) plays a master role in regulating the stomatal conductance and is recruited to initiate adaptive responses to drought (Zhu 2002). A number of genes participate in the ABA biosynthesis and responses, such as the key ABA biosynthesis gene 9-*cis*-epoxycarotenoid dioxygenase (NCED) and the ABA receptor phosphatase 2C (PP2C) (Tuteja 2007; Fujita *et al.* 2011). Further, ABA interacts with other phytohormone pathways in drought responses. Jasmonic acid (JA) was showed to act in concert with ABA and was implicated to function as a positive regulator of stomatal closure (Creelman and Mullet 1995; Suhita *et al.* 2004; Munemasa *et al.* 2007). In *Arabidopsis*, the loss-of-function of cytokinin-related genes, *AHP2*, *AHP3*, and *AHP5*, resulted in up-regulation of ABA-responsive genes (Nishiyama *et al.* 2013). Other phytohormones, such as salicylic acid (SA), auxin (AUX), ethylene (ETH), brassinosteroid (BR), and gibberellin (GA), are implicated in drought responses, although the respective roles are not as clear as that of ABA (Saruhan, *et al.* 2012; Iwata *et al.* 2013; Uga *et al.* 2013; Skirycz *et al.* 2011; Dubois *et al.* 2013). Besides protein-encoding genes, microRNA (miRNA), and long non-coding RNA (lncRNA) are involved in regulation of plant drought responses (Zhang *et al.* 2014; Ding *et al.* 2013; Ferdous *et al.* 2015).

Most studies examined regulation of gene expression on drought through comparing plants at well-watered conditions and plants at high levels of drought stress at a selected time after exposure (Kakumanu *et al.* 2012; Kizis and Pagès 2002). However, under natural conditions, plants progressively suffer increasing drought stress, and both physiological and underlying transcriptional responses to drought vary at different levels of drought stress. Dynamic responses to progressively increasing drought conditions have been characterized in a few crop species. Recently, a time-series transcriptome study of *Arabidopsis* on drought stress provided an overview of temporal responses to drought and demonstrated that distinct responses at different time points for many genes (Bechtold *et al.* 2016). Transcriptomic dynamics of multiple points during the drought stress and recovery have been investigated in tomato, finding that repression of genes related to photosynthesis, cell proliferation and cell cycle, and chromatin associated processes, as well as activation of genes in many pathways such as ABA biosynthesis upon drought (Iovieno *et al.* 2016). In maize, gene expression patterns of maize seedlings were examined at multiple time points (3 and 6 days) after drought treatment and 1-day after water recovery, finding that genes involved in photosynthesis and hormone biosynthesis were regulated upon both drought and re-watering (Zhang *et al.* 2018). Recently, transcriptomic profiling of leaves, ears, and tassels at multiple stages of maize development found drought imposition caused more transcriptional changes in leaves and ears than changes in the tassel (Danilevskaya *et al.* 2019). Here, we analyzed time-series transcriptomic data of maize seedlings at well-watered and drought conditions. Drought responsive genes were identified and characterized with respect to their variation of copy number in diverse *Zea mays* lines, their genetic association with drought resistance, and their relationship to expression of small RNAs quantified using the same set of samples. Our results demonstrated the value of time-series transcriptomic profiling data, which deepens our understanding of dynamic gene responses upon progressive drought stress in maize.

## MATERIALS and METHODS

### Plant materials and drought treatments

Tissue samples from B73 seedlings prepared in a previous study that examined small RNA expression were used for RNA-Seq in this study, and detailed growth conditions and drought treatment were described (Zheng *et al.* 2019). Briefly, for drought treatment, the pots were watered when the seedlings emerged and then were subjected to drought stress up to 10 days after withholding water (DAW). At 10 DAW, plants were divided into two groups: Group 1 plants continued under drought stress (DS), and Group 2 plants were watered. Samples were then prepared from both groups at day 11. The above-ground tissues of five seedlings were collected for each sample and immediately frozen in liquid nitrogen for extraction of total RNA and quantification of abscisic acid (ABA). For each DAW from 3 to 10, plants with well-watered were collected as the control. Total RNA was isolated from the harvested samples using the TRIzol reagent (Invitrogen). Quantification of ABA was performed as described using a liquid chromatography-mass chromatography system (López-Carbonell and Jáuregui 2005).

### mRNA sequencing experiment

Illumina TruSeq library preparation kit was used to prepare sequencing libraries for sequencing on a HiSeq2000 to produce paired-end 2x100bp reads at the Berry Genomics (Beijing, China). Two biological replicates per time point (DAW) per treatment were performed.

### mRNA data process

The software Trimmomatic (version 0.32) (Bolger *et al.* 2014) was used to trim the adaptor sequence of mRNA raw reads. The parameters used for the trimming is: “ILLUMINACLIP:trimming_db:3:20:10:1:true LEADING:3 TRAILING:3 SLIDINGWINDOW:4:13 MINLEN:40”. The trimming adaptor database includes the sequences: adaptor1, AGATCGGAAGAGCGTCGTGTAGGGAAAGAGTGTA; adaptor2, AGATCGGAAGAGCACACGTCTGAACTCCAGTCAC. Only the paired reads both of which are at least 40 bp after trimming were retained for further analyses.

Paired-end reads were aligned to the B73 reference genome (B73Ref3) (Schnable *et al.* 2009) using STAR (version 2014-05-15) (Dobin *et al.* 2013). The main parameters of STAR are “–alignIntronMax 100000 –alignMatesGapMax 100000 –outFilterMismatchNmax 2 –outFilterMismatchNoverLmax 0.02”. A confident alignment of each read is required to be at a single alignment locus with at least 50 bp match, 98% minimal identity, 98% minimal coverage, and maximal 5 kb genomic spanning length. And then the coordinates of genes from the filtered gene set were compared to each alignment to determine the number of reads per gene.

### Identification and clustering of significantly drought-responsive genes

A statistical test, implemented with DESeq2 (Love *et al.* 2014), was performed to test the null hypothesis that no interaction between time (DAW) and treatment for a given mRNA in the model of “mRNA ∼ DAW + Treatment + DAW x Treatment”, where the treatment has two levels: drought stress (DS) and well-watered (WW). Average 2 reads per sample were required for the genes to be subjected to the statistical test. The DAW and Treatment interaction effects were examined separately on the Day (DAW) ranging from DAW 3 to 10, from DAW 3 to 6, from DAW 6 to 8, and from DAW 8 to 10, to obtain the whole-course, early, middle, and late drought-responsive genes (DRGs), respectively. The Benjamini-Hochberg method was used to account for multiple tests to control the false discovery rate (FDR) (Benjamini and Hochberg 1995). The FDR cutoff was set at 5% to identify DRGs.

The estimates of mRNA expression for samples at both the DS condition and the WW condition at DAW 3-10 through the model were used to perform the mRNA clustering analysis with the R package “mclust” (Scrucca *et al.* 2016). The model selection was based on BIC values. The model “VVV” was selected because it had the highest BIC value (Fig S1). With the “VVV” model, ten major components were identified. These ten components were then used to determine the 10 clusters of DRGs.

### Identification of differentially expressed genes between DS and RW

To test the null hypothesis that no differences existed in gene expression between DS and re-watering (RW) groups at the 11^th^ day, a generalized linear model for the read count of each gene implemented in the DESeq2 package (version 1.4.5) was performed. The FDR approach was used to account for multiple tests (Benjamini and Hochberg 1995) and the fold change (FC) in expression per gene between DS and RW was also determined by DESeq2. The criteria with FDR smaller than 5% and absolute log2(FC) larger than 1 were used as a cutoff for differential expression.

### Enrichment analysis

The enrichment analyses were performed to determine if a certain type of category, such as a member of gene ontology, transcription factor families, or small RNA (sRNA) functional families, is over-represented in a selected group of genes/sRNAs. To account for the biases due to read depth that influences the selection of members in a given group, the resampling method in the GOSeq enrichment test (Young *et al.* 2010) using total reads across all the samples of a certain mRNA/sRNA as the bias factor was applied to all the enrichment analyses in this study. The gene ontology database was extracted from the file “ZmB73_v3.gene2go.txt” downloaded from AgriGOv2 (http://systemsbiology.cau.edu.cn/agriGOv2/). GO terms with *p*-values < 0.01 were considered as significantly enriched terms and the top 20 GO terms for each subset were used to plot heatmaps with the “pheatmap” R package. The transcription factor database was downloaded from grassius.org as of 10/7/2014 and a chi-square test was used to detect the enrichment of TF families under drought stress (*p*-value < 0.05).

### Cluster analysis of genes in phytohormone pathway

Genes associated with 8 phytohormones were downloaded from MapMan Mapping database (Zm_B73_5b_FGS_cds_2012, https://mapman.gabipd.org/mapmanstore). The log_2_ fold changes of gene expressions of DS/WW from 3 to 10 DAW were used for hierarchical cluster analysis in each phytohormone pathway, which includes genes involved in the biosynthesis and its regulation, and then the heatmap plot for clustered genes was generated using the “pheatmap” package in R.

### Identification of significantly negative correlated sRNA-gene pairs

Small RNAs were obtained from our previous study using the same plant samples (Zheng *et al.* 2019). To identify sRNA-gene pairs, the online tool “psRNATarget” (Dai and Zhao 2011) was used to predict the targeted mRNAs of sRNAs with default parameters (-penalty for G:U pair 0.5, -seed region 2-13, -mismatches allowed in seed region 2, -HSP size 19, -penalty for opening gap 2, -penalty for extending gap 0.5, -translation inhibition range 10-11) (Dai and Zhao 2011). For sRNAs, a cutoff with at least 1.0 expectation was used to filter the prediction results of sRNA-gene pairs and for known miRNAs, a cutoff with at least 1.5 expectation was used to filter the prediction results of miRNA-gene pairs. In addition, sRNAs that target more than 30 mRNAs were excluded. We then perform correlation tests to test the null hypothesis that sRNA-gene correlation is no less than 0 using log-transformed values of normalized read counts adjusted by adding 1. The Benjamin-Hochberg method was used for multiple test correction (Benjamini and Hochberg 1995).

### Degradome analysis to identify drought-responsive mRNA-sRNA pairs

Two sets of raw degradome reads, including B73 ear degradome (SRP025172) and B73 root/leaf degradome under low nitrate condition (SRP018376) were downloaded from NCBI Sequence Read Archive (SRA). After adaptor sequences and low-quality sequencing reads were removed with Trimmomatic, clean reads were used to identify cleavage sites on the basis of the B73 cDNA sequences (5b+). CleaveLand version 4.0 was then performed for degradome analysis with default parameters (Addo-Quaye *et al.* 2009). sRNA-mRNAs pairs identified by degradome analysis were then compared with pairs predicted by psRNATarge to provide evidence for genes targeting by sRNAs.

### Copy number variation analysis of DRGs

Copy number variation (CNV) genes were obtained from a previous *Zea mays* CNV study, including both maize and teosinte (Swanson-Wagner *et al.* 2010) and then merged with DRGs to identify drought-responsive CNV genes on the early, middle, late and whole-course drought stages. Enrichment analysis was then performed on drought-responsive CNV genes to find whether they were enriched on different drought stages. To confirm the enrichment of drought-responsive CNV genes, a Pearson correlation between read counts per line of each gene and library size per line was calculated using whole genome sequencing data of 269 maize inbred lines, a subset of maize 282 diverse lines (Tian *et al.* 2011). For a given gene with no or low sequence variation, read counts per line of the gene should be highly correlated with the library size per line, saying that the correlation between gene read counts and library sizes should be as high as close to 1. Sequence variation of a gene, particularly CNV, among lines would result in a lower correlation. We therefore used this correlation as an index to examine sequence variation or CNV, including present and absent variation (PAV), of a gene among these diverse maize lines.

### GWAS analysis of DRGs in a maize population

The genotypes used for GWAS analysis were generated from a maize panel consisting of 368 diverse inbred lines, and a total of 525,105 high-quality SNP markers with minor allele frequency no more than 0.05 were identified (Li *et al.* 2013). The population structure and kinship matrix of 368 maize inbred lines were also generated from previous GWAS work of the same population (Fu *et al.* 2013). Based on survival rates after severe drought stress, drought tolerance levels per line of this maize panel at the seedling stage were reported in the previous study (Liu *et al.* 2013). GWAS was performed using Tassel5.0 (Bradbury *et al.* 2007) under the MLM model by using the population structure as covariance. From GWAS results, SNPs with *p*-value small than 1E-4 were deemed as the top association markers, and genes (from 1 kb upstream to 1 kb downstream of each gene) that contained the top association markers were identified as candidate genes.

We also determined GWAS *p*-values for all genes. Among all SNPs on the region of a gene (the transcription region plus 1 kb upstream and 1 kb downstream of a gene), the smallest *p*-value of all was used to represent the drought associated *p*-value (DAP) of the gene.

### Data availability

Sequencing raw data are available from Sequence Read Archive: PRJNA483231. Codes were shared at Github (github.com/liu3zhenlab/manuscripts/tree/master/mRNA_Drought_2020). Supplemental material available at figshare: https://doi.org/10.25387/g3.11918463.

## Results

### Time-series transcriptomes of maize seedlings under drought and well-watered conditions

**Figure 1:**
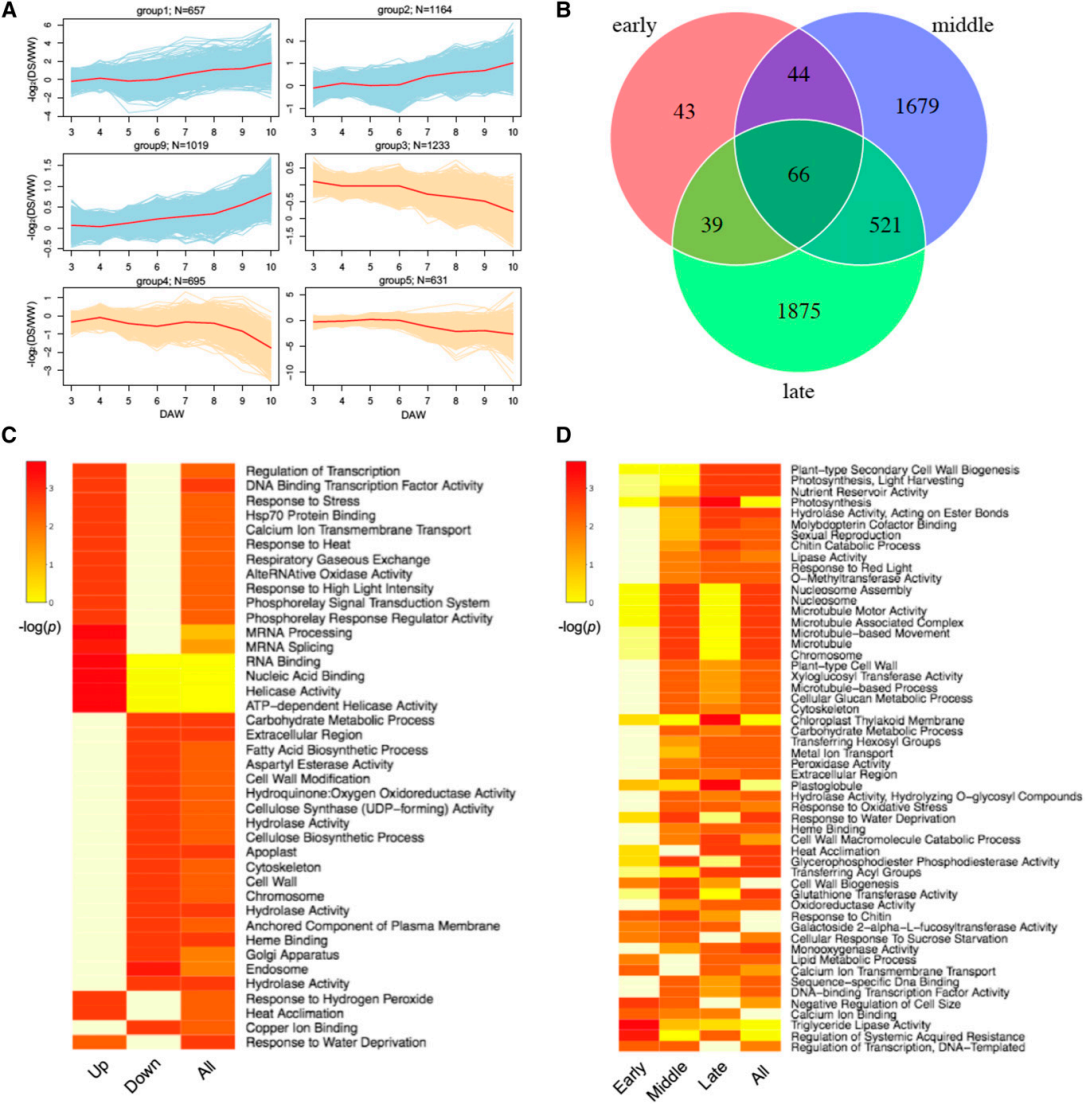
Overview of drought-responsive genes (DRGs). (A) Major clustering groups of DRGs. Each curve represents the ratio of average gene expression of DS to WW with a log2 transformation (y-axis) *vs.* days after withholding water (DAW) (x-axis). The red curve represents the mean values from all genes in a cluster. The N number on the top of each clustering group indicates the number of DRGs in this group. (B) Venn Diagram of DRGs at different drought stages. (C) GO enrichments of up, down-regulated DRGs, and all DRGs identified from the whole-course (3-10 DAW) analysis. (D) GO enrichments of early, middle, late DRGs, and all DRGs.

We performed message RNA (mRNA) transcriptomic analysis of maize inbred line B73 seedlings under drought stress (DS) at multiple time points, 3 to 10 days after withdrawing watering (DAW), in comparison with corresponding seedlings under the well-watered (WW) condition (Fig S2, Fig S3). Re-watering was performed at 10 DAW on some seedlings from the DS group, enabling comparison between re-watering and DS samples at 11 DAW to understand regulation of gene expression on re-watering. Totally 36 seedling samples were collected for transcriptomic analysis, including two biological replicates per time point (DAW) per treatment. RNA sequencing of seedling samples resulted in 19.4-37.3 million pairs of 2x100 bp paired-end reads per sample. Approximately 99% reads were retained after adaptor and quality trimming, and, on average, 82.7% clean reads were confidently mapped to a single location on the B73 reference genome (B73Ref3) (Table S1). The same samples were subjected to small RNA sequencing (Zheng *et al.* 2019). To identify genes exhibiting responses to the drought treatment, the hypothesis that no effects from the interaction between time points for DAW, from 3 to 10 days, and treatments, DS and WW, on gene expression was tested for each gene. The tests resulted in 8,626 genes with significant DAW-treatment interactive effects at the level of the 5% false discovery rate (FDR) and were designated as drought-responsive genes (DRGs) (Table S2). Principal Component Analysis (PCA) of the expression of DRGs showed DS samples after 6 DAW were clustered and separated from earlier DS samples, WW samples, and the re-watering sample, indicating a large proportion of variation in expression was derived from differential expression between DS and WW samples after 6 DAW (Fig S4). The DRGs were identified without using RNA-Seq from the re-watering seedling samples, which, however, were clustered near WW samples, implying the expression of DRGs was largely recovered upon re-watering. To understand the regulation pattern of each DRG over the course of drought treatment, DRGs were clustered based on the expression ratios of DS to WW samples at each time point, which resulted in 10 clusters. Six major clusters, consisting of 6,539 DRGs, were categorized into two groups, up-regulated on DS (up-regulated DRGs or uDRGs) and down-regulated on DS (down-regulated DRGs or dDRGs), which contained 2,840 and 3,699 genes, respectively (Figure 1A, Fig S5). Gene ontology (GO) enrichment analysis indicated that genes with the regulatory activity (*e.g.*, nucleic acid binding) are overrepresented in uDRGs, while genes involved in metabolic processes and cell structure establishment (*e.g.*, lipid metabolic process and nucleosome) are overrepresented in dDRGs (Figure 1C).

The progressive drought treatment results were divided into three stages: early stage (from 3 to 6 DAW), middle stage (from 6 to 8 DAW), and late stage (from 8 to 10 DAW), based on changes of leaf water contents upon drought stress (Fig S6). Statistical tests to examine DAW-treatment interactions were performed to identify the DRGs at each stage. Because almost all DRGs identified separated from three stages overlap with 8,626 DRGs identified from the whole drought course, 3 to 10 DAW, further analyses only focused on these 8,626 DRGs. Of them, 192 early DRGs, 2,310 middle DRGs, and 2,501 late DRGs were identified at the level of the 5% FDR (Figure 1B). GO enrichment analysis showed that different sets of GO terms are overrepresented in DRGs of the three drought stages (Figure 1D, Table S3). Of early DRGs, genes associated with transcription regulation pathway are enriched, whereas genes in stress-related pathways, such as pathways related to water stress and oxidative stress, were overrepresented at both middle and late DRGs. Of late DRGs, photosynthesis-related genes are also highly enriched. Some early, middle, and late DRGs overlap. Among 4,268 non-redundant DRGs found in any of three stages, 66 were detected as DRGs at all the three stages and 25.8% of them (17/66) were transcription factors (TFs). We examined transcription factors (TFs) in DRGs at three drought stages. In total, 497 DRGs are TFs from 52 families, including 8, 33, 32 TF families at early, middle, and late stages, respectively (Table S2, Fig S7). Genes from two TF families, ZmWRKY and ZmZIM (now designated as the TIFY family), previously documented to play roles in drought tolerance (Chen *et al.* 2012; Ren *et al.* 2010; Vanholme *et al.* 2007; Ye *et al.* 2009), were enriched in the DRGs in all three stages (p-value < 0.01) (Table S4), and genes of ZmNAC, a known drought-responsive TF family (Mao *et al.* 2015; Shiriga *et al.* 2014), was enriched in early and middle DRGs. Other TF families that were specifically enriched in the DRGs of certain drought stages include ZmGRAS, ZmEREB, and ZmMYB, which were specifically enriched in early, middle, and late DRGs, respectively.

### Expression profiling of re-watered drought stressed plants

**Figure 2:**
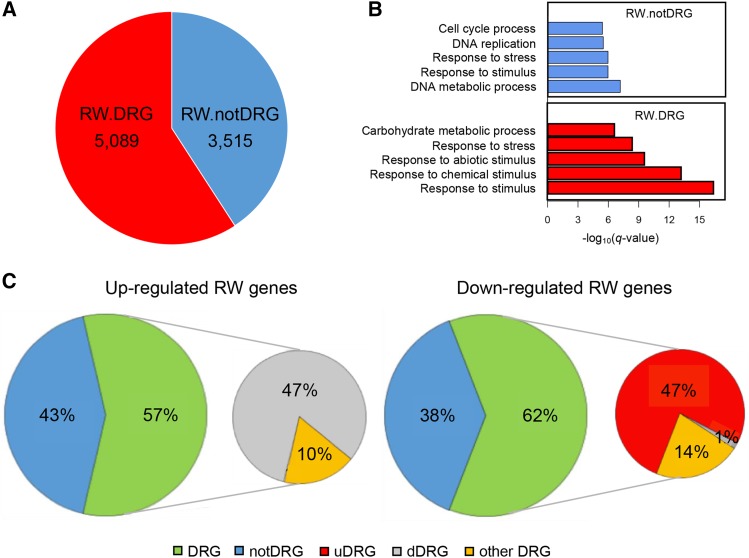
Differential expression between RW and DS samples at DAW 11. (A) Genes that overlap and did not overlap with DRGs are represented by RW.DRG and RW.notDRG, respectively. (B) Top 5 GO terms enriched in differentially expressed genes. (C) Percentages of up- and down-regulated genes upon re-watering. The “notDRG” represents drought unaffected genes. Other DRG stands for ungrouped genes in the cluster analysis.

A comparison of gene expression was also performed between two additional seedling groups, DS at 11 DAW and re-watering (RW) samples from the 11^th^ day, which was subjected to 10 days drought stress and re-watered at 10 DAW. Leaf relative water content (RWC), soil water content (SWC) and leaf relative electrical conductivity (REC), a measure of cell damage (Wang *et al.* 2008; Bajji *et al.* 2002), were quantified for WW, DS and RW samples to evaluate the degree of drought stress. Note that the experiment and data of these measurements was previously described (Zheng *et al.* 2019). Levels of all these measurements of re-watering plants were close to the levels of WW plants (Fig S6), indicating that plants were recovered from drought stress. Comparison between DS at 11 DAW and RW samples identified 8,604 differentially expressed (DE) genes, of which 5,089 overlapped with DRGs and 3,515 genes were not significantly affected by drought (drought unaffected genes) (Figure 2A). GO enrichment analysis of 5,089 DE-overlapping DRGs showed that 4 of the top 5 most significant GO terms were related to stress response, whereas 3,515 DE-overlapping drought unaffected genes were enriched in DNA metabolic and cell cycle pathways (Figure 2B). Of DE-overlapping DRGs, 2,327 and 1,768 were up- and down-regulated upon re-watering, respectively. It is interesting but not surprising that most DE genes exhibited the opposite regulation upon re-watering as compared to their regulation on DS (Figure 2C), indicating that expression of most DRGs recovered to near the levels at the well-watered condition.

### Expression changes of phytohormone-related DRGs during progressive drought stress

**Figure 3:**
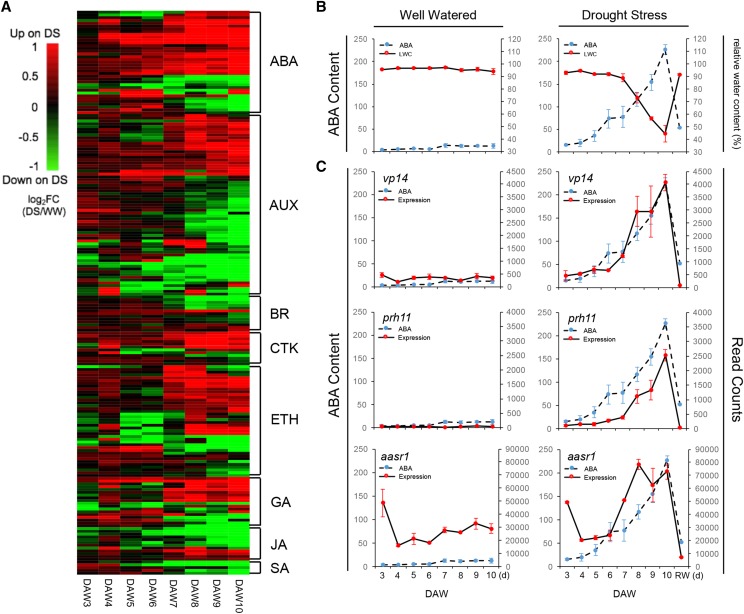
DRGs in hormone pathways. (A) Heatmap of expression regulation of phytohormone genes on drought. The log_2_FC value is the log_2_ of the ratios of DS to WW in the mean expression. (B) ABA content and leaf relative water content (RWC) changes under both WW and DS conditions. (C) ABA content and gene expression changes of three ABA related genes: *vp14*, *prh11*, and *aasr1*.

In total, 202 DRGs were related to eight phytohormone pathways (Table S5), including ABA, AUX, BR, CTK, ETH, GA, JA, and SA. DRGs associated with each hormone pathway were clustered based on their expression patterns (Figure 3A). Most DRGs related to ABA, CTK, ETH, and GA were up-regulated under drought stress, particularly at the late drought stages, whereas most DRGs in AUX, JA and SA pathways were down-regulated at middle or late drought stages.

ABA levels were quantified for both DS and WW samples. The ABA content increased from 15.4 to 226.0 (ng/g) in DS plants from 3 to 10 DAW, of which leaf water content decreased. In contrast, the ABA content remained at low concentrations, from 4.2 to 12.9 (ng/g) over the same period in WW plants (Figure 3B). When DS plants were re-watered at 10 DAW, the ABA content was restored to a level similar to WW plants. To understand underlying alternation of ABA synthesis related genes under drought stress, the time-series expression profiles of three ABA-related genes *Vivparous14* (*vp14*), *Protein phosphatase homolog11* (*prh11*) and *Abscisic acid stress ripening1* (*aasr1*), were examined. The expression of *vp14* (GRMZM2G014392), encoding a 9-*cis*-epoxycarotenoid dioxygenase (NCED) that is the rate-limiting enzyme for ABA biosynthesis (Tan *et al.* 1997), was highly correlated with ABA contents. The expression of *prh11* (GRMZM2G159811), which encodes the core ABA receptor ZmPP2C (Xiang *et al.* 2017; Wang *et al.* 2018), increased in parallel but apparently lagged behind with ABA accumulation (Figure 3C). The ABA downstream gene, *aasr1* (GRMZM2G136910), which was reported to be highly induced by ABA (Zhang *et al.* 2019), increased in expression with the increase of ABA.

### Enrichment tests of early and middle DRGs via CNV and GWAS analyses

**Figure 4:**
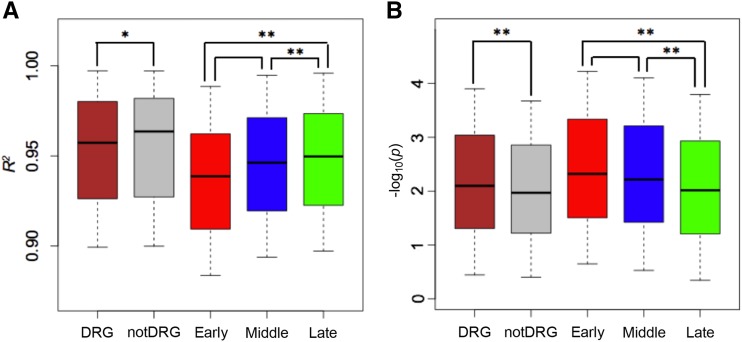
DRGs, CNV, and GWAS. (A) Boxplot of proportion of variation in read counts per gene of 269 maize lines explained by sequencing depths, or library size (*R*^2^). The lower value of *R*^2^, the higher level of sequence variation among diverse lines is. The “notDRG” stands for drought unaffected genes. Early, Middle, and Late represent DRGs in each drought stage, respectively. (B) Boxplot of log_10_ transformed drought association *p*-values (DAPs) from drought GWAS using 367 maize lines. (* *P* < 0.05, ** *P* < 0.01).

A previous study revealed 3,805 copy number variation (CNV) genes among maize lines and lines from their ancestor relative teosinte (Swanson-Wagner *et al.* 2010). Genes with copy number variation (CNV) have been reported to be overrepresented in abiotic stress responses (Maron *et al.* 2013; Knox *et al.* 2010; Xu *et al.* 2006a). Enrichment analysis indicated these CNV genes were not overrepresented in all DRGs (*p*-value = 0.13, Table 1). However, separate enrichment tests on early, middle, and late DRGs showed that CNV genes were enriched in early and middle DRGs, and the frequency of early DRGs with CNV was higher than that of middle DRGs (0.098 *vs.* 0.076). The smaller *p*-value of middle DRGs is probably due to the higher statistical power gained from a higher number of middle DRGs as compared to early DRGs. Independent examination using whole genome sequencing data of 269 maize inbred lines (a subset of maize 282 lines) (Tian *et al.* 2011) indicated that means of correlations between sequencing depths per gene and total sequencing depths across these inbred lines were different among DRG groups. The correlation between read depths of a gene with total sequencing depths reflects the level of sequencing variation of the gene among diverse lines. Late DRGs and drought unaffected genes showed similar higher levels of correlations as compared with early and middle DRGs, and early DRGs exhibited the lowest correlation (Figure 4A). The results indicated that early and middle DRGs possessed a higher level of sequencing variation, likely contributed by CNV, than late DRGs and drought unaffected genes.

**Table 1:** Enrichment of CNV genes in early, middle, late DRGs

DRGs	P-value of the $\chi^{2}$ test	Frequency of CNV genes in DRGs	Frequency of CNV genes in other genes
All	0.13	0.060 (518/8,626)	0.055 (970/17,524)
Early	0.019	0.098 (19/193)	0.057 (1469/25,957)
Middle	5.12E-05	0.076 (175/2,310)	0.055 (1279/23,840)
Late	0.23	0.062 (156/2,501)	0.056 (1277/23,649)

Reanalyzing of a previous genome-wide association study (GWAS) used the trait of surviving rates of 367 diverse maize inbred lines after the drought treatment identified 84 genetic variants associated with drought tolerance (*p*-value < 1e-4), and 37 candidate genes (Table S6) (Liu *et al.* 2013). Among them, 5 genes were associated with the highest significant GWAS peaks (*p*-values < 1e-5), including *ZmVPP1* (GRMZM2G170927), the bZIP transcription factor *ZmbZIP23* (GRMZM5G821024) and the H(+)-ATPase coding gene (GRMZM2G035520). Of 37 candidate genes, 16 were DRGs in the present study (Table 2). The most significant GWAS candidate gene in 16 candidate genes is a homolog of a plasma membrane ATPase (H(+)-ATPASE 11, AHA11) in *Arabidopsis*. To examine the association levels of early, middle, and late DRGs, as well as drought unaffected genes with drought resistance, the lowest value of GWAS *p*-values of all SNPs on or around a gene was used to represent the drought association *p*-value (DAPs) of the gene. Thus, a DAP value per individual gene was assigned. The average DAPs of DRGs were significantly lower than that of drought unaffected genes. In addition, early and middle DRGs, particularly early DRGs, exhibited stronger associations than late DRGs (Figure 4B). The result suggested that early responsive DRGs are more likely to be associated with drought adaptation and some of them are likely to play roles in drought tolerance.

**Table 2:** GWAS candidate DRGs

GeneID	Adjusted p-value of DRG	Regulation on drought	DAP from GWAS	Gene Name	Function Annotation	Arabidopsis homologs
GRMZM2G035520	3.70E-05	dDRG	1.01E-06	HA11	H(+)-ATPase 11	AT5G62670
GRMZM2G132212	9.49E-03	others	1.09E-05		Protein kinase family protein with leucine-rich repeat domain	AT5G25930
GRMZM2G036134	4.56E-02	dDRG	1.15E-05		Eukaryotic aspartyl protease family protein	AT3G54400
GRMZM2G092165	1.67E-04	uDRG	1.51E-05		Nucleic acid-binding, OB-fold-like protein	AT2G40780
GRMZM2G087635	1.48E-08	dDRG	2.19E-05		Transmembrane amino acid transporter family protein	AT1G80510
GRMZM2G107196	1.07E-03	dDRG	2.22E-05	pdlk1	pyruvate dehydrogenase kinase	AT3G06483
GRMZM2G159477	1.91E-02	uDRG	2.81E-05		Alpha/beta hydrolase related protein	AT2G40095
GRMZM2G106250	4.43E-23	uDRG	2.82E-05		3-hydroxyacyl-CoA dehydrogenase family protein	AT3G15290
GRMZM2G026147	2.96E-05	others	4.23E-05	EXPL1	Expansin-like A1	AT3G45970
GRMZM2G018059	1.55E-03	dDRG	4.26E-05		U-box domain-containing protein kinase family protein	AT2G45910
GRMZM2G178787	9.72E-03	uDRG	5.19E-05	PK2B	Protein kinase 2B	AT2G02800
GRMZM2G001930	2.29E-02	others	5.73E-05	bHLH149	Basic helix-loop-helix (bHLH) DNA-binding family protein	AT1G32640
GRMZM2G130332	2.22E-03	dDRG	6.13E-05		diaminopimelate epimerase family protein	AT3G53580
GRMZM2G086163	3.74E-03	others	7.08E-05		Heavy metal transport	AT5G03380
GRMZM2G375116	1.43E-08	uDRG	8.99E-05		Potassium transporter family protein	AT5G14880
GRMZM5G885529	1.67E-02	uDRG	9.58E-05		unknow	AT5G18440

### Pairs of drought-responsive small RNAs and genes

**Figure 5:**
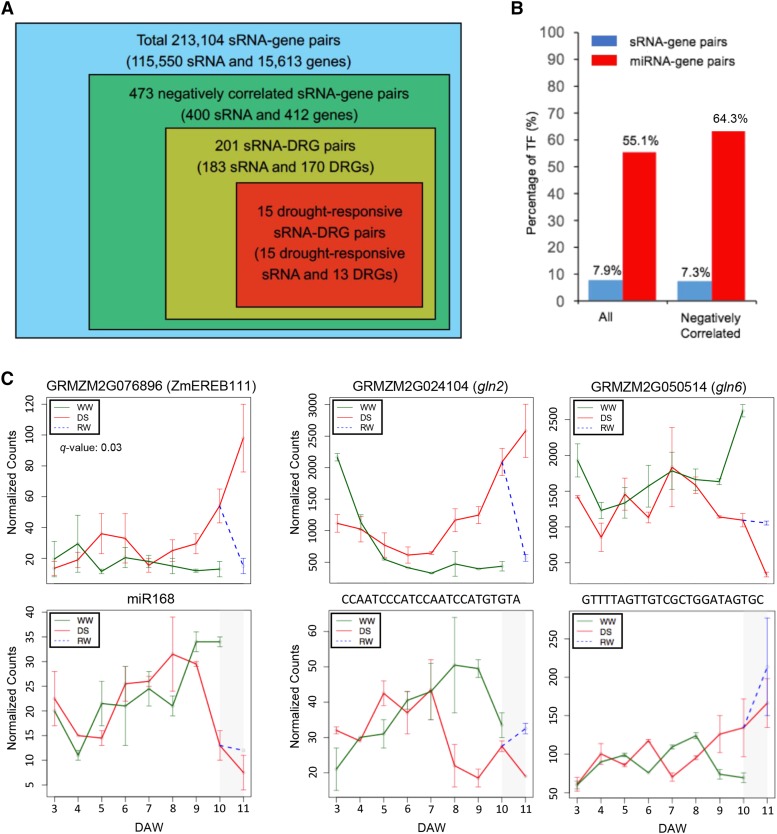
Pairs of drought-responsive sRNA and DRGs. (A) Summary of sRNA-gene pairs identified in this study. (B) Enrichment of TFs in sRNA-gene pairs and miRNA-gene pairs. (C) Time-series changes in expression of both sRNAs and genes of three pairs.

Our previous study examined expression profiles of small RNAs (sRNAs) of the same seedling samples used for genes (mRNAs) expression in this study (Zheng *et al.* 2019). Time-series expression data of both sRNAs and genes enabled identification of sRNA-gene pairs with significant correlations in expression. A prediction for sRNA targeted genes from 688,121 sRNAs identified 213,104 sRNA-gene pairs, which included 115,550 sRNAs targeting 15,613 genes. For each pair, normalized read counts per sample of each sRNA and each gene were then used to test the hypothesis that no negative correlation between a sRNA and a gene from 3 to 10 DAW, as well as from both drought and re-watering expression data at 11 days. As a result, we found 473 pairs, including 400 sRNAs and 412 genes, with significant negative correlations using the 5% FDR (Figure 5A, Table S7a). Of 400 unique sRNAs, 39 could be functionally categorized. Briefly, 17, 11, 9, and 2 sRNAs were annotated as miRNAs, ribosome RNA derived sRNAs, small nucleolar RNA (snoRNA) derived sRNAs, and transfer RNA derived sRNAs, respectively. Both the miRNA and the snoRNA derived sRNA were enriched in these 400 sRNAs (χ^2^ test, both p-values < 2.2e-16). The result indicates that miRNA and snoRNA derived sRNAs are two important sRNA groups regulating genes involved in seedling development during the drought treatment and/or drought responses, the two factors driving sRNA/gene expression changing in our experiment.

In total, 201 sRNA-gene pairs exhibited negative correlations in expression between sRNAs (N = 183) and DRGs (N = 170) (Table S7b). One of reported drought responsive TFs, *ZmEREB111* (GRMZM2G076896), which involved in ABA regulation, was targeted by the stress responsive miR168 and up-regulated on DS (Figure 5C) (Zhou *et al.* 2010; Ding *et al.* 2013). Most DRGs (64.3%) targeted by miRNAs are TFs, supporting the previous finding that miRNAs function largely through pairing with TFs (Rhoades *et al.* 2002; Samad *et al.* 2017) (Table S7c, Figure 5B). Briefly, 31 TFs in 16 TF families were in these sRNA paired DRGs, including 6 ZmEREB, 3 ZmNAC, 2 ZmbZIP, 2 ZmWRKY, and 1 ZmbHLH. All these TF families have been reported to be involved in responses to drought stress in maize, rice, or *Arabidopsis* (Olsen *et al.* 2005; Liu *et al.* 2014; Chen *et al.* 2012; Abe 2003). Besides TFs, two glutamines synthetase genes, the early uDRG, GRMZM2G024104 (*glutamine synthetase2*, *gln2*), and the late uDRG, GRMZM2G050514 (*glutamine synthetase6*, *gln6*), were paired with two unknown sRNAs, CCAATCCCATCCAATCCATGTGTA and GTTTTAGTTGTCGCTGGATAGTGC, respectively (Figure 5D). Based on the drought GWAS result, *gln2* and *gln6* were the two genes showing the highest levels of association with drought tolerance among six genes in the glutamine synthetase family (Table S8). Of 201 sRNA-DRG pairs, 15 sRNAs were also drought-responsive (Table 3). These 15 pairs were further examined by using degradome RNA sequencing datasets, which provides evidence for cleavage sites on mRNAs through sRNA targeting. Two degradome datasets were analyzed, including degradomes from B73 ear samples (Liu *et al.* 2014) and degradomes from B73 root/leaf under low nitrate condition (Zhao *et al.* 2013). One drought-responsive sRNA-late DRG pair was evidenced with the B73 ear degradome data (Fig S8A), whereas five drought-responsive sRNA-DRG pairs, including *gln6* and the *CBF1* orthologous gene, were identified in B73 degradomes under nitrate stress (Fig S8B). The results supported the hypothesis that some drought-responsive sRNA-DRG pairs involved in multiple abiotic stress conditions.

**Table 3:** Negatively correlated drought-responsive sRNA-DRG pairs

GeneID	Gene Symbol	DRG regulation on drought	Drought-responsive sRNA	Correlation between sRNA and DRG
GRMZM2G096764	NA	uDRG	AAAGTTTATCTCGCGAGATTGGAA	−0.674
GRMZM2G099297	NA	dDRG	AATAAAAAGAAACGGAGGGAG	−0.614
GRMZM2G085249	*aprl5*	uDRG	AATGCTTCGAAGGACGAAGGACTT	−0.726
GRMZM2G108416	NA	uDRG	ACTAAAACAAACATGATTTAACGT	−0.477
GRMZM2G080912	CBF1	uDRG	AGAGTGTTCTGTAAAGGACAGAGA	−0.727
GRMZM2G386273	*bzip55*	others	ATAAACTACGGTATTGTCATAACT	−0.505
GRMZM2G140150	NA	dDRG	CCAATACACATGGATTGGATGGGA	−0.631
GRMZM2G034947	NA	uDRG	CCATCCCCATCCCCTAATGGAGGA	−0.684
GRMZM2G034947	NA	uDRG	CCCGTCCCCGTTTACCCGTCGGGG	−0.708
GRMZM2G030862	NA	dDRG	CTCTCTATTTATAGAGGAGGGGGG	−0.623
GRMZM2G034947	NA	uDRG	GAATTCCCCGCGGGGAATCGGGGA	−0.706
GRMZM2G050514	*gln6*	dDRG	GTTTTAGTTGTCGCTGGATAGTGC	−0.56
GRMZM2G059428	*NAC53*	others	TCGGCTGCTAGTTTCTACAGTGGC	−0.472
GRMZM2G023110	NA	dDRG	TGGATCAATAAGAGTGGTAGCT	−0.729
GRMZM2G179294	NA	others	TTAAGTTGTTATTTGGTGACGA	−0.523

## Discussion

Drought is a major constraint for production of all staple crops, and molecular mechanisms of drought tolerance are complicated. Our time-series transcriptomics study that used seedlings of a maize inbred line at controlled drought environments attempted to reduce confounding factors and identified DRGs and their responsive patterns along progressive drought imposition. Overall, we found that regulatory activities and stress defensive pathways were induced, whereas enzymatic activities related to synthesis of cellular components were suppressed upon drought stress, which is consistent with the suppression of plant growth under drought stress. Time-series transcriptomic data enable us to examine dynamic gene responses over progressive drought stress, to characterize DRGs with respect to CNV, relevance to drought tolerance, and interactions with sRNAs. Our study provides valuable transcriptomic data for comparisons with gene drought responses in other tissues, under different drought environments, and in other plant species, as well as candidate genes that potentially play roles in tolerance to drought, and other abiotic stresses.

Time-series gene expression results showed that different functional groups of genes were regulated at early, middle, and late drought stages. Some early DRGs (N = 43) were specific in drought responses at the early stage, while many (N = 66) showed constitutive responses at all the stages. The former includes three TFs two of which are from the NAC family, and the latter has 13 TFs including four WRKY and three bHLH TFs. These TFs, particularly the genes that were only responded at the early stage, are likely to be in the pathways signaling osmotic stress created by water content changes and regulating downstream genes to initiate adaptively physiological responses. We compared the genes showing constitutive responses with a transcriptomic study (Miao *et al.* 2017) using leaves, ears, and tassels from multiple stages of adult plants under natural drought treatments and the irrigation treatment as the control. Two third of these 66 genes overlapped drought-responsive genes on adult leaves, and only 15–16% overlapped drought-responsive genes on ears or tassels, which indicated that the same tissue type preserved more similar drought responses than different tissues did. In the middle and late drought stages, more DRGs were identified in annotated stress-related pathways, such as oxidative stress, indicating plant stress defensive pathways have been initiated. Approximately 60% of these middle and late DRGs were drought responsive in adult leaves or other tissue types from the spatiotemporal drought study (Miao *et al.* 2017). Photosynthesis-related genes were highly enriched in late DRGs, implying the perturbation of photosynthesis at the severe drought condition. The impacts of drought on photosynthesis in diverse tissues have been found through both physiological and transcriptomic studies (Chen *et al.* 2016; Miao *et al.* 2017; Zhang *et al.* 2018). Here we showed that the process was largely affected at the late drought stage of seedlings. Drought responses of maize under field condition showed that the up-regulated genes were highly enriched in abiotic response pathways and the down-regulated genes were highly enriched in DNA metabolic processes, which is consistent with drought responses under seedling stages in our study (Danilevskaya *et al.* 2019). Several drought responsive transcription factor genes detected in ear under field condition, such as *ZmMADS32* (GRMZM2G105387), *ZmMADS9* (GRMZM2G005155), *ZmMADS7* (GRMZM2G097059) and *ZmYAB7* (GRMZM2G102218), can also be found through studying seedling transcriptome under progressive drought stress (Danilevskaya *et al.* 2019).

Our analysis indicated that early and middle DRGs contain higher levels of genetic variation, and are more likely to be associated with drought tolerance. Given the potential roles of early and middle DRGs in drought tolerance or susceptibility, the high levels of genetic variation, including gain and loss of gene function or genes *per se*, might be the consequence of adaptation of maize lines to changing growth environments. Plant stress responses generally come at the cost of fitness (Berens *et al.* 2019). Stress responsive genes that are in the pathways interacting with environmental stresses could be absent at certain scenarios to reduce fitness cost, resulting in presence and absence variation (PAV), a particular type of CNV. This is supported by the finding that genes exhibiting CNV are overrepresented in the pathways of stress responses in multiple crop species, such as maize, soybean, and grape (Swanson-Wagner *et al.* 2010; McHale *et al.* 2012; Cardone *et al.* 2016). Early DRGs contain 19 CNV genes encoding proteins of multiple families: NAC transcription factors (GRMZM2G167018 and GRMZM2G079632), EF hand calcium-binding protein (GRMZM2G474755), zinc finger like proteins (GRMZM2G070797 and GRMZM2G101664), Polyketide cyclase/dehydrase (GRMZM2G019246), dehydrases (GRMZM2G019246), and hypersensitive induced reaction protein (GRMZM2G070659), etc. Homologs of some genes have been shown to be involved in drought tolerance. For example, overexpression of a maize NAC transcription factor (ZmNAC111) increased water-use efficiency and improved drought tolerance of maize seedlings (Mao *et al.* 2015). For another example, ectopic expression of an EF hand calcium-binding protein (MtCaMP1) from *Medicago truncatula* in *Arabidopsis* increased seedling survival rates under both drought and salt stresses (Wang *et al.* 2013). EF-hand family proteins were documented to participate in signaling biotic and abiotic stresses by perception and transduction of calcium signals. Therefore, the early responsive EF hand calcium-binding gene (GRMZM2G474755) might function in sensing drought stress and transducing signals for drought responses. In maize, the contribution of these early CNV DRG genes to drought tolerance is unknown. However, since CNV of these genes exists among maize lines and many of these genes are likely to be dispensable, natural variation, ectopic expression, and knockouts can be used for functional examination of their drought responsive roles. We should note that the drought relevance of early and middle DRGs does not exclude the importance of some late DRGs in drought tolerance, which could be functionally essential, and, therefore, are highly conserved in sequence among examined lines.

In our experiment, to find the interaction between sRNAs and genes under drought, we quantified the accumulation of both sRNAs and gene transcripts from the same samples. Our assumption is that sRNAs, particularly miRNAs, target genes and trigger mRNA degradation, thereby reducing the accumulation of their cognate gene transcripts. Such post-transcriptional regulation by sRNAs would be detected through analyzing sRNA-gene correlations with sRNA and mRNA transcriptomic data from multiple samples. However, the analysis would not be successful if the post-transcriptional regulation of gene expression is not simply the one-to-one sRNA and mRNA interaction. For example, from our prediction of sRNA-gene pairs, on average, a gene is targeted by 7.4 sRNAs. Many genes are targeted by more than 10 sRNAs. The lack of one-to-one relations reduces the possibility to detect interactions based on accumulation levels of sRNA and genes. In plants, sRNAs also can function to repress translation of mRNAs (Song *et al.* 2019; Zhou *et al.* 2010), which would not be detected using the correlation method with transcriptional data. Although there are drawbacks, the analysis found 201 candidate pairs of sRNAs and DRGs, among which some are likely to be responsible to drought tolerance. For instance, an early DRG, *gln2* suggested to be targeted by a sRNA was up-regulated upon drought and the GWAS result showed *gln2* has a high level of association with drought tolerance. The phenotypic association was supported from a rice study in which overexpression of the rice *gln2* ortholog *GS1-3* enhanced drought resistance (Cai *et al.* 2009; Singh and Ghosh 2013). MiRNAs as gene regulators are expected to participate in the regulation of drought responsive genes. Among all the negatively correlated sRNA-gene pairs, there were 17 miRNA-gene pairs consisted of 11 known miRNAs and 15 genes. Of these genes, 64.3% (9/14) genes were transcription factors, including 3 SBP TFs (*ZmSBP8*, *ZmSBP23*, *ZmSBP30*), which function to promote phase transitions and flowering time, targeted by a well-known drought responsive miRNA, *miR156* (Eldem *et al.* 2012; Kantar *et al.* 2011; Liu *et al.* 2008; Wu and Poethig 2006; Zhou *et al.* 2010), and 2 GRF TFs (*ZmGRF6* and *ZmGRF7*), which function on leaf and cotyledon development, targeted by another drought responsive miRNA, *miR396* (Eldem *et al.* 2012; Liu and Yu 2009; Sun 2012). Moreover, we also identified an up-regulated drought responsive TF (*ZmEREB111*) that was negatively correlated with *miR168* (Wei *et al.* 2009). Collectively, these previous data supported that our results provide valuable miRNA-gene pairs for further characterization.

We performed comparative analyses of time-series changes for ABA content and their related genes on both DS and WW conditions to elucidate how phytohormone profiles change in response to drought stress. ABA is well-known to stimulate short-term drought responses such as stomatal closure (Zhang and Davies 1987; Kim *et al.* 2010). In our results, 37 DRGs are in ABA-related pathways. Of them, 20 were up-regulated on DS and the majority of these up-regulated DRGs were highly positively correlated with ABA contents, including multiple ABA biosynthesis key genes. The results indicated that these ABA biosynthesis genes were co-regulated to increase ABA production under drought stress. A number of ABA-responsive genes were up-regulated on drought, presumably due to the increase of ABA contents. Overexpression of some ABA responsive genes, such as *ABF1* and *ABF2*, enhanced plant drought tolerance in other plant species (Yoshida *et al.* 2015; Fujita *et al.* 2005; Kim *et al.* 2004). In our study, we detailed the regulation of genes in the ABA pathway by drought and provided the candidates for further genetic analysis. Moreover, 1, 15 and 7 ABA-related DRGs showed their responses at early, middle, and late drought stages, respectively, implying that ABA-mediated drought responses were vigorously activated from the middle drought stage. The levels of JA and SA were also related to drought response (Creelman and Mullet 1995; Miura and Tada 2014). Application of exogenous JA and SA can both enhance drought tolerance in plants (Anjum *et al.* 2011; Kang *et al.* 2013). However, the levels of JA and SA were reported to be negatively regulated by ABA under drought stress (Yasuda *et al.* 2008; Nakata *et al.* 2013). We found 6/12 genes from the JA pathway and 4/5 genes from the SA pathway are DRGs and they were all down-regulated on drought (Table S5). In JA pathway, 3 of 4 lipoxygenase genes (*lox4*, *lox12*, *lox13*), which encoded the key enzymes for JA biosynthesis (Vick and Zimmerman 1983; Vick and Zimmerman 1984), were down-regulated under drought stress and a JA signaling gene (*ts1*), which functions on sex determination of maize, was also found to respond to drought stress (Acosta *et al.* 2009). In maize plants at the adult stage, many lipoxygenase genes were activated upon stress imposed by natural drought conditions (Danilevskaya *et al.* 2019). The distinct drought responses of JA genes might be related to differential tissue responses or different stress environments. Jasmonates are known to be repressors for cell-cycle genes, and, through that, it was hypothesized to suppress cell division and ear growth (Danilevskaya *et al.* 2019). However, from our expression data, both genes in JA and cell-cycle pathways were largely down-regulated on drought. Therefore, additional examinations are needed to reveal the regulation roles of JA in drought responses. Methyl salicylate (MeSA), the bioactive SA conjugates, is normally absent in plants but is dramatically induced upon abiotic or biotic stress (Loake and Grant 2007). MeSA is synthesized by SA carboxyl methayltransferase (SAMT), using the methyl donor S-adenosyl-l-Met and carboxylic acid containing substrates (Zubieta 2003). Three maize genes (GRMZM2G039993, GRMZM2G063438, GRMZM2G116966) homologous to the SAMT genes in *Arabidopsis* and rice were identified as DRGs and two of them were down-regulated on drought stress (Koo *et al.* 2007; Xu *et al.* 2006b; Zhao *et al.* 2010). Our results supported the negative association between the accumulation of ABA and the overall expression of the JA and SA pathways, which would result in low levels of JA and SA accumulation, in the context of drought stress in maize seedlings (Urano *et al.* 2017).
